# Magnitude of adverse neonatal birth outcomes and associated factors among babies born from adolescent mothers in East Hararghe zone, Oromia region, Eastern Ethiopia

**DOI:** 10.1186/s12884-026-09793-9

**Published:** 2026-08-07

**Authors:** Gadise Belete, Haymanot Mezmur, Yared Tadess, Wubshet Nebiyu, Tefera Belsty, Merhawi Gebremedhin

**Affiliations:** 1 School of Public Health, Haramaya University, Harar, Ethiopia; 2 Department of Human Anatomy, School of Medicine, Haramaya University, Harar, Ethiopia

**Keywords:** Adverse birth outcome, Adolescent mothers, Pregnancy in adolescent, Ethiopia

## Abstract

**Background:**

The pregnancy rate among adolescents is increasing, particularly in developing countries, with higher adverse health outcomes. There are limited studies conducted to assess the adverse neonatal birth outcomes of adolescent pregnancy and associated factors for newborns in Ethiopia.

**Objective:**

To assess the adverse neonatal birth outcomes and associated factors among babies born to adolescent mothers in Hospitals of East Hararghe Zone, Eastern Ethiopia from 21/01/ 2023 to 20/01/2024.

**Methods:**

A cross-sectional study was conducted on 532 medical recordsof adolescent delivery mothers using a single population and double -proportion formula to identify factors associated with adverse neonatal birth outcomes. Data was collected using Kobo, EPI INFO version 3.2, and analyzed using STATA version 17, Bivariate and multivariable binary logistic regression, and Hosmer-Lemeshow tests, with significance reported at a value < 0.05.

**Result:**

Data were extracted from 532 single-tone chart mothers. The prevalence of adverse birth outcomes in neonates born to adolescent mothers was 37.22(95% CI: 33.1–43.1). Spontaneous vaginal delivery (AOR = 0.35,95% CI:0.19–0.65), Low Hgb (AOR = 1.71, 95% CI: 1.04–2.81), APGAR scores first minute < 7 (AOR = 0.55, 95% CI:0.45–0.66), APGAR scores five minute < 7 (AOR = 1.11,95% CI:1.00-1.22), induced for labor (AOR = 3.5, 95% CI: 1.11–11.11), poor maternal nutrition (AOR = 0.107, 95% CI: 0.018–0.63), and mother with Eclampsia (AOR = 6.3,95%CI:1.74–22.75), had a significantly higher risk for adverse birth outcomes.

**Conclusion:**

The overall prevalence of adverse neonatal outcomes reported in this study was relatively high compared to findings reported in previous studies, such as those in Nigeria and Turkey. Adolescent mothers with spontaneous vaginal delivery, low Hgb levels, eclampsia, nutritional status, induced delivery during delivery, and Apgar scores in the 1st and 5th minutes were all significantly associated with adverse neonatal outcomes. Addressing factors like maternal nutrition, access to prenatal care, and proper delivery management could help reduce these adverse outcomes and improve overall maternal and neonatal health for adolescent mothers.

**Supplementary Information:**

The online version contains supplementary material available at https://doi.org/10.1186/s12884-026-09793-9.

## Background

Adolescent pregnancy is defined as to pregnancy occurring in girls aged 10 and 19 [1, 2]. Between 2015 and 2020, approximately 15% of women gave birth before the age of 18 ,with 90% of these births occurring in low a middle-income countries. In developing countries, adolescent pregnancy remains high with about one in three adolescent girls become pregnant. Globally, nearly one in five adolescent girls has given birth. Adolescent pregnancy is a major global public health issue, with approximately 21 million girls between the ages of 15 and 19 become pregnant each year in both developed and developing nations [3].

The highest rate of teenage pregnancy in the world is sub–Saharan Africa [4].In developing countries, the average age of expectant mothers is lower than in Western countries, and approximately 11% of all births worldwide occur among adolescent mothers [5] .

Due to the complexity and impact of the problems involved, adolescent pregnancies represent a public health problem. Adolescent pregnancy is strongly associated with adverse neonatal outcomes such, as prematurity, low birth weight, low Apgar scores, and neonatal mortality, especially in mothers under 15 years of age. Furthermore the pregnancy rate among adolescents is increasing, especially in developing countries, with higher adverse health outcomes [4]. The few studies conducted in Ethiopia to assess the factors associated with adverse neonatal birth outcomes among adolescent pregnancies. In addition, all of the existing studies focus on comparisons between adolescent and adult pregnancies rather than on estimating the magnitude of adverse neonatal outcomes among adolescents [4, 6]. So, this study aims to assess the magnitude of adverse neonatal birth outcome and associated factors among babies born from adolescent mothers in East Hararghe Zone, Oromia Region, Eastern Ethiopia.

## Methods and materials

### Study setting, design, population, and period

A hospital-based retrospective cross-sectional study was conducted in the Eastern Hararghe Zone of Ethiopia. Data from all adolescent mothers who delivered in hospitals during one year from 21/01/2023, to 20/01/2024, were reviewed. The Eastern Hararghe zone borders Bale to the southwest, West Hararghe to the west, Dire Dawa to the north, and the Somalia region to the east. Based on July 2023 population projection data, this zone has a total population of 4035,797, of which 2041,217 are males and 1994,580 females. With an area of 17,935.40 square kilometers, East Hararghe has a population density of 151.87. While 216,943 or 8.27% are urban, another 30,215 or 1.11% are pastoralists. The East Hararghe zone has 21 woredas and 2 towns, 582 health posts,115 health centres,158 clinics, and 8 hospitals. Of these, there are 4 general and 4 primary hospitals. All health facilities in the zone provide delivery service to the surrounding community.

### Inclusion and exclusion criteria

Adolescent mothers aged 10 to 19 years with complete delivery and birth outcome data from selected hospitals were included. Adolescent mothers with incomplete birth documentation were excluded from the study.

### Sample size determination

#### The sample size for the first objective

The sample size for the first objective is calculated using the single proportion formula n= (z (α/2)) ^2^ p (1-p)/d ^2^ by considering the following assumptions. Where n = the minimum sample size, z (α/2) = the desired 95% confidence interval (1.96). *P* = 34.9% of adverse fetal outcomes in adolescent mothers from the previous study [6]. d = margin of error (5%). Using the above information, *P* = 34.9%, the initial sample size was calculated to be 349. After calculating the initial sample size using the single-population proportion formula, a design effect of 1.5 was applied to adjust for the sampling technique. The final sample size was calculated by multiplying the initial sample size (349) by 1.5, resulting in a total of 524 participants.

#### The sample size for the second objective

The double population proportion formula is used to calculate the sample size for adverse birth outcomes of adolescent mothers and associated factors using Epi info based on the following assumptions; 95% confidence level, 80% power, and an exposed-to-unexposed ratio of 1 (Table 1).

**Table 1:** Sample size calculation for the factors associated with adverse birth outcomes (P2 and AOR)

Associated factors and adverse birth outcome	% outcome to exposed	% outcome to unexposed	AOR	Sample size	Reference
Premature birth	61.4%	38.5%	2.5	166	[7]
Residence	57.2%	44.7%	1.6	532	[4]
No ANC follow up	12%	4.5%	2.8	472	[4]

Finally, from the sample size calculation for the first and second objectives, the largest sample for this study is *n* = 532

#### Sampling procedure

**Fig. 1:**
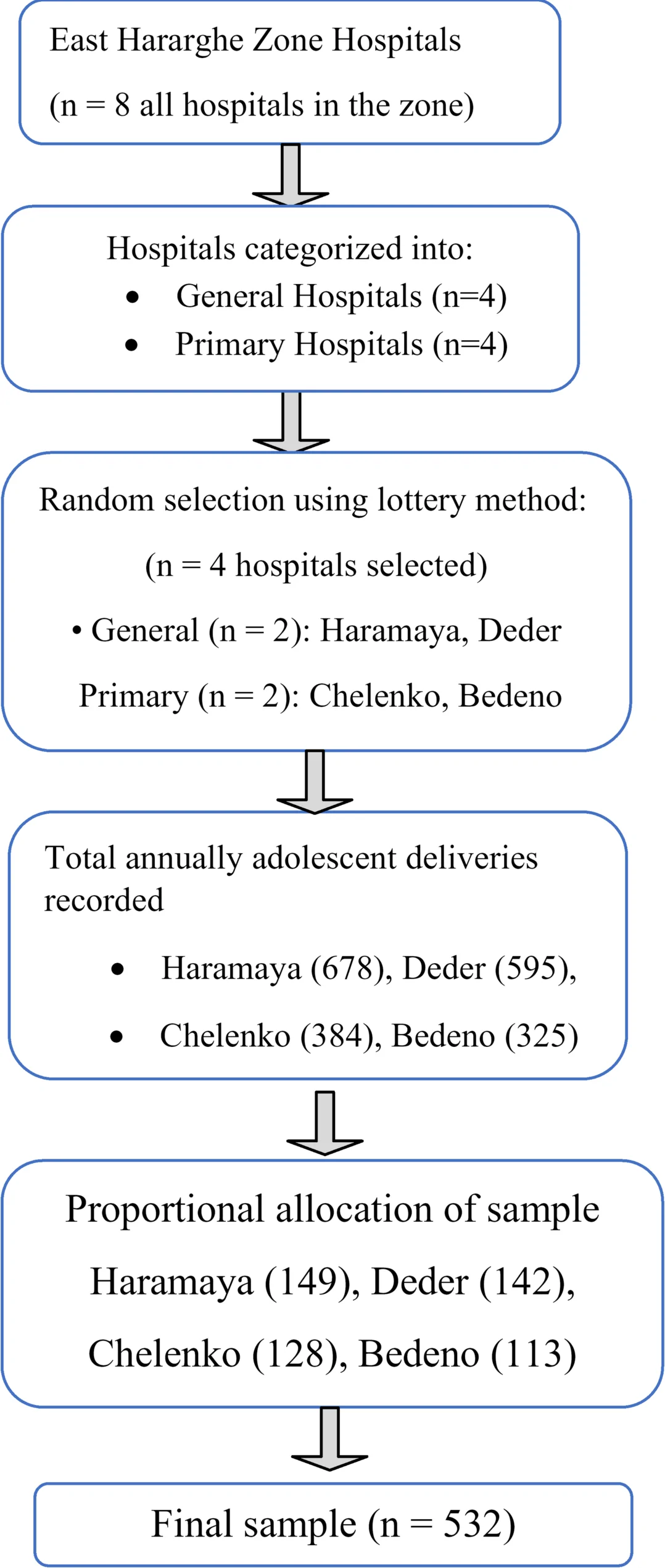
Sample size allocation for study on adverse neonatal birth outcome among adolescent mothers in East Hararghe zone hospitals, Eastern, Ethiopia,2024

Multi-stage sampling methods were used to select representative study participants. Initially, hospitals in the zone were categorized into General and Primary hospitals. Four hospitals were selected using simple random sampling by the lottery method: two General hospitals (Haramaya Hospital and Deder Hospital) and two Primary hospitals (Chelenko Hospital and Bedeno Hospital). The sample sizes were proportionally allocated based on each hospital’s annual adolescent delivery coverage (Fig. 1). Finally, participants were selected from each hospital using simple random sampling. The delivery registration books from 21/01/2023 to 20/01/2024 served as the sampling frame.

### Data collection tools and procedures

The data was collected using a questionnaire prepared in English after reviewing similar literature in the field area and related documents [4, 6, 8, 9]. The STROBE Cross-Section Guidelines were used to ensure the quality, transparency, and completeness of the study [10].

The data collectors were Bachelor of Science (BSc) midwives from selected hospitals who had experience handling variables in the checklist and recording maternal and child health data. Data collectors were trained for two days before data collection by the principal investigator and experienced supervisors. Training effectiveness was assessed through pre/post-tests. Two supervisors were also enrolled. The data were collected by reviewing the maternity documents of adolescents who came from the hospital during the study period from January 21, 2023, to January 20, 2024. Before data extraction began, the documents were checked for completeness using a checklist. Data collectors were instructed to write card numbers on the checklist when collecting data so that they could be reviewed if problems or errors were found. The completed checklist was checked daily for completeness by data collectors, supervisors, and principal investigators.

### Variables

The dependent Variables is Adverse neonatal birth outcome. Residence, marital status, educational level, occupation, low ANC follow-up, poor nutrition intake, anaemia, hypertension, malaria, HIV, delivery mode, current obstetric complication, and gestational age are the independent variables.

### Operational definitions

Adverse neonatal outcome: An adverse birth outcome is defined as the occurrence of at least one of the following adverse outcomes in a baby born in a hospital: the need for resuscitation after delivery, a low Apgar score, fetal death during labor, low birth weight, congenital anomalies, immediate neonatal death, birth asphyxia, admission to the neonatal intensive care unit (NICU), preterm delivery, and neonatal jaundice within 24 h of birth [11].

Low birth weight: Babies who are born weighing less than 2.5 kg (2,500 g) [12].

Neonatal Death: Death of a live newborn baby within the first 28 days of birth [13].

Pregnancy-induced hypertension Preeclampsia, a pregnancy-specific disorder characterized by hypertension (≥ 140/90 mm Hg) and proteinuria (≥ 300 mg in a 24-hour urine) [14].

Preterm delivery: Babies born alive before 37 weeks of pregnancy are completed. Less than 259 days since a woman’s first day of the last menstrual period [15].

### Data quality control

To ensure the quality of the data, the availability of variables was checked from hospital documents before the actual data collection was carried out. To clarify the question, conditions, and the requirements for completion the checklist. The principal investigators supervised the data collectors’ daily activities and checked the completeness of the filled questionnaire. Data completeness and consistency were checked before and after data entry by calculating frequencies for each variable.

### Data processing and analysis

The data collected with the Kobo tool were checked for completeness and then exported to STATA version 17 for analysis. Descriptive statistics, including simple frequencies and cross-tabulations, were carried out. Categorical variables were recategorized and categorized, while continuous variables were summarized as means ± standard deviation (SD) and numbers (percentages). Adverse birth outcomes were measured using a dichotomous response (yes/no). Logistic regression was used to identify factors associated with adverse neonatal outcomes in adolescent pregnancies.

Bivariate analysis was performed to assess the association between adverse neonatal outcomes and each independent variable. Variables with a P-value less than 0.25 in binary regression and those that are significant based on existing literature were included in the multivariable binary logistic regression. The model fit was assessed using the Hosmer-Lemeshow test, where a value more than 0.05 indicates a good fit. Multicollinearity was assessed using the Variance Inflation Factor (VIF). Variables with a P-value of less than 0.05 were considered to be statistically significant predictors of adverse birth outcomes in adolescent deliveries. The results were reported as odds ratios with 95% confidence intervals.

## Results

### Sociodemographic characteristics

A total of 532 delivery charts were reviewed, with a response rate of 100% meeting the study’s eligibility criteria. The average age of the study participants was 17 years, the minimum and maximum ages were 13 and 19 years, respectively. In terms of age, the majority of participants, 510(95.86%) were between 16 and 19 years old, while only 22(4.14%) participants were in the 13–15 age group. In terms of marital status, 485(91.17%) were married. A smaller number of 46(8.65%) participants (8.65%) were single, and only 1(0.19%) participant was divorced. Residential data show that 400(75.19) participants lived in rural areas, while 132(24.81%) participants lived in urban areas. Regarding education, 357(67.11%) participants (67.11%) had no formal education, while 175(32.89%) participants had formal education.

Finally, in terms of employment, garniture was the most common occupation with 315(59.21%) people. This was followed by housewives, with 176(33.08%) participants, and students, with 41 (7.71%) participants.

### Health -seeking behavior and obstetric history

Of a total of 386 adolescent mothers who had undergone follow -up, almost all had fewer ANC check-ups during their pregnancy, 378(97.93%), and 8(2.08%) of them were followed up more than four times. About 380(72%) adolescent mothers received the TT vaccine during their pregnancy, and the remaining 145(27.61%) were unvaccinated. 407(76.5%) of adolescent mothers were counselled and screened for malnutrition in this study, 68(17%) were moderately malnourished. In this study,407(76.5%) adolescent mothers took iron supplements during their pregnancy, and 266(65.35%) of those who took iron for less than three months. About 62 (11.51%) adolescent mothers had complications during pregnancy; of these, 20(3.79%) had preeclampsia, followed by APH 17(3.20%) and 10(2.89%) premature membrane rupture. 450 (84.59%) of the mothers delivered spontaneously, followed by 82(15.41%) by cesarean section, and 47(8.83%) delivered before 37 weeks of pregnancy.

Of all deliveries, 82 (15.41%) of study participants had complications during delivery,29 (35.37) were labor disorders, followed by eclampsia 27(32.93%), 18(21.95%) malpresentation /malposition of the fetus, and 9.76% did not develop complications during labor. According to this study, adolescents whose labor lasted longer than 16 h from the start accounted for 29(5.45%) of all births.

### Medical history of adolescent mothers

Of all adolescent mothers who tested their haemoglobin levels, 109(20.49%) of the mothers were anaemic. About 520(97.74%) of the adolescents were screened for HIV Virus and 2 (0.53%) of them were positive for the virus. Almost all 530 (99.62%) participants were screened for pregnancy-induced hypertension, 78(14.66%) of them were screened for malaria, and 1 positive case was found.

### Newborn outcomes

The majority of mothers (90.98%) delivered their child in a full-term pregnancy after 37 weeks of gestation. More than half of newborns in the study 55.45% were born male, about 467(87.78%) had a cephalic presentation, 9% were breech, and 3% had a shoulder injury. 90% 483(90%) of the babies were born alive, 208(39.10%) of the newborns were admitted to NICU, and 92.57% recovered (Table 2).

**Table 2:** Characteristics of newborns in the study of adverse birth outcomes among adolescent deliveries in hospitals of Eastern Ethiopia,2024

Variables	Category	Frequency	Per cent %
Gestational Age	< 37 weeks	47	8.83
	> 37 weeks	485	90.98
Fetal presentation	Breech	48	9.02
	Cephalic	467	87.78
	Shoulder	17	3.2
sex of newborn	Female	236	44.55
	Male	296	55.45
birth outcome	Alive	482	90.60
	Dead	50	9.40
birth weight	< 2500gram	78	15.09
	> 2500gram	454	85.34
1st minute Apgar score	< 7	85	15.97
	>=7	446	83.83
5th minute Apgar score	< 7	54	10.15
	>=7	478	89.85
NICU admission	Yes	145	27.79
	No	387	71.65

### Proportion of adverse birth outcome

**Fig. 2:**
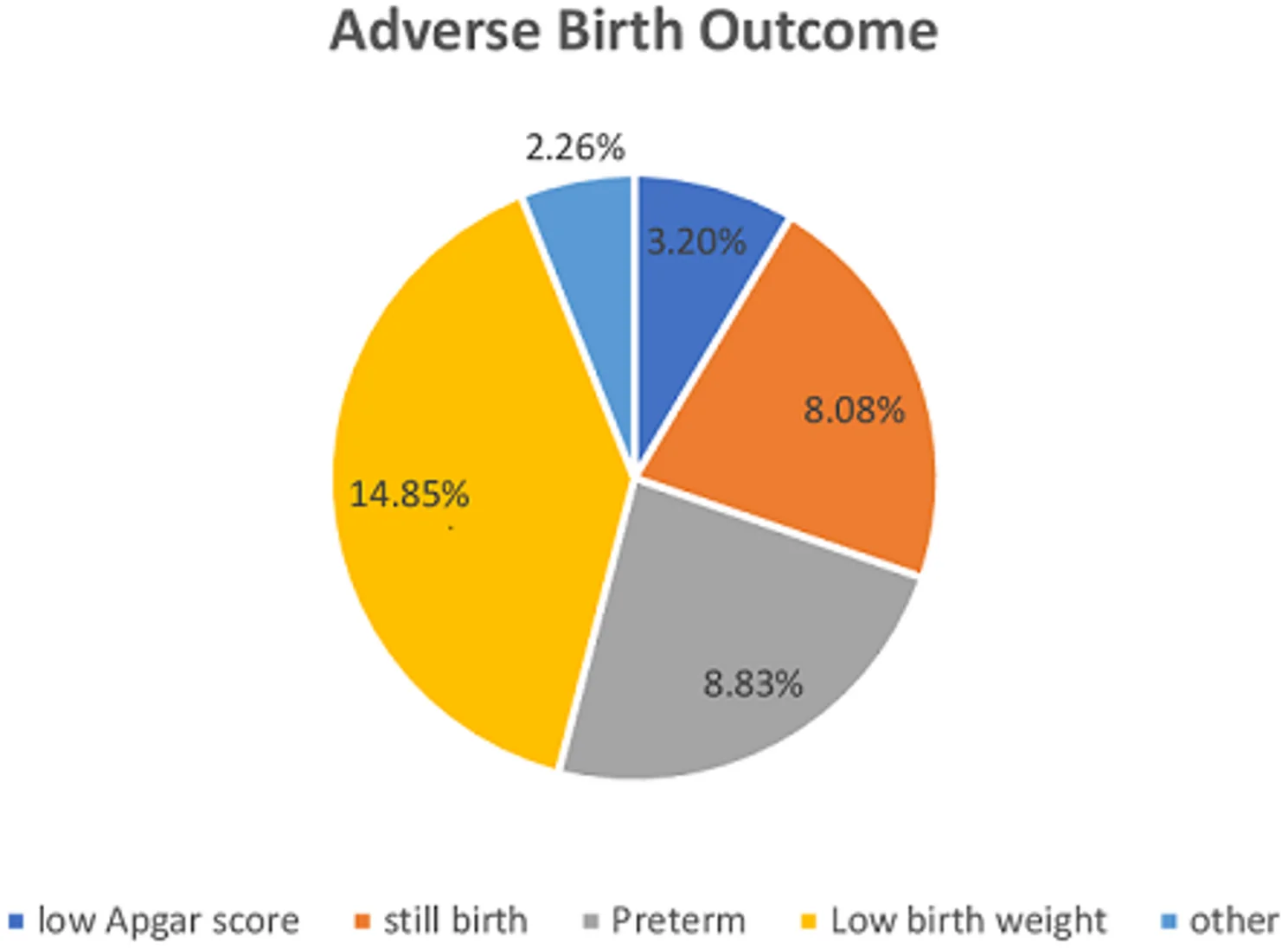
Proportion of adverse birth outcomes among adolescent deliveries in Hospitals of Eastern Ethiopia,2024

The result of this study showed that the prevalence of adverse birth outcomes in adolescent deliveries was 198 (37.22%). Of these 79(14.85%) had low birth weight, 47(8.83%) had Preterm birth, 43(8.08%) had a stillbirth, and 17(3.20%) had low Apgar score (Fig. 2).

### Factors associated with adverse birth outcomes

A binary logistic regression model was used to identify factors associated with adverse birth outcomes in adolescent delivery. Based on the bivariate model, factors found to be significantly associated with adverse birth outcomes were marital status, educational level, nutritional status, mode of delivery, a maternal complication such as APH, preeclampsia, eclampsia, fetal malpresentation, obstructed labor, induction, Apgar score in the 1st and 5th minute, maternal haemoglobin level, iron supplementation, mode of delivery, TT vaccine and occupation as significant (Table 3).

**Table 3:** Bivariate and Multivariate analysis of adverse birth outcomes among adolescent deliveries in Hospitals of Eastern Ethiopia,2024

Variables	Category	Adverse Birth outcome		COR 95%, CI	AOR 95%, CI
		yes	No		
Marital status	Single	35(76.09%)	11(23.91%)	1	
	Married	252(51.96%)	233(48.04%)	0.42(0.22–0.77) *	0.79(0.26–2.35)
Educational level	No formal education	177(49.58%)	180(50.42%)	1	
	formal education	110(62.86%)	65(37.14%)	1.76(1.22–2.55) *	1.41(0.81–2.47)
Mode of delivery	SVD	34(35.42%)	62(64.58%)	0.47(0.29–0.76)	0.35(0.19–0.65) **
	Cesarean Section	252(57.93%)	183(47.02%)	1	
Occupation	Farmer	163(51.91%)	151(48.09)	1	
	Housewife	96(54.24%)	81(47.02%)	1.02(0.71–1.5)	0.85(0.49–1.49)
	Student	28(68.29%)	13(31.71%)	3.25(1.65–6.41)*	1.41(0.24–4.553)
Nutritional status	Normal	165(49.70%)	167(50.30%)	0.51(0.30–0.87)	0.107(0.018–0.63)**
	Moderate AcuteMalnutrition	43(63.24%)	25(36.26%)	1	
Last high level	8-11 mg/dl	190(62.30%)	115(37.70%)	1.92(1.33–2.77)	1.71(1.04–2.81)**
	12-13 mg/dl	97(42.73%)	130(57.27%)	1	
TT vaccine	Yes	131(24.62%)	249(47.42%)	0.92(0.79–1.06)*	1.05(0.83–1.33)
	No	63(12%)	82(15.61%)	1	
APGAR 1st minute	< 7	58(10.90)	27(5.1%)	0.16(0.11–0.23)	0.55(0.45–0.66)**
	> 7	91(33.46%)	181(66.54%)	1	
APGAR 5th minute	< 7	52(9.77%)	7(1.31%)	0.96(0.87–0.99)	1.11(1.00-1.22)**
	> 7	151(28.38%)	327(61.46%)	1	
Baby Presentation	Cephalic	249(53.32%)	218(46.68%)	0.96(0.53–1.75)	1.35(0.55–3.31)
	Breech	26(54.17%)	22(45.83%)	0.94(0.50–1.74)	
	Shoulder	12(70.59%)	5(29.41%)	1.92(0.73–5.09) *	0.48(0.103–2.23)
Induction	Yes	11(68.75%)	5(31.25%)	4.11(1.46–11.5)	3.51(1.11–11.11)**
	No	186(36.12%)	240(46.60%)	1	
APH	Yes	15(78.95%)	4(21.05%)	3.32(1.08–10.15) *	2.74(0.56–13.26)
	No	272(53.02%)	241(46.98%)	1	
Eclampsia	Yes	23(76.67%)	7(23.33%)	2.96(1.25–7.03)	6.3(1.74–22.75) **
	No	264(52.59%)	238(47.41%)	1	
Preeclampsia	Yes	15(75.00%)	5(25.00%)	2.64(0.94–7.39) *	3.07(0.65–14.5)
	No	272(53.13%)	240(46.88%)	1	
Iron supplementation	< 3month	97(23.83%)	169(41.52%)	0.81(0.58–1.12) *	0.78(0.51–1.21)
	> 3month	43(10.56%)	98(24.07%)	1	
Obstructed labor	Yes	10(37.04%)	17(62.96%)	0.39(0.15–1.01) *	0.6(1.08–2.38)
	No	33(60%)	22(40%)	1	

*COR *Crude odd ratio, *AOR *Adjusted odd ratio

**=*p*-value < 0.05 *=*p*-value < 0.25

## Discussion

Adolescence, defined by the World Health Organization (WHO) as the period between the ages of 10 and 19, is a critical period in human physical and psychosocial development in which an individual transitions from an immature state to a mature reproductive state [16]. Adolescent pregnancy was associated with an increased risk of adverse pregnancy outcomes such as low birth weight, stillbirth, preterm birth, maternal anaemia, eclampsia, and maternal death [17, 18].This study assessed the adverse outcomes of neonatal adolescent pregnancies in Eastern Ethiopia. This study found a high prevalence of adverse neonatal birth outcomes in adolescent pregnancies. Adolescent mothers who had eclampsia, low Hgb levels, induced labor, had poor maternal nutrition, and those who delivered by spontaneous delivery had a significantly higher risk of adverse birth outcomes.

According to this study, the magnitude of adverse birth outcomes among adolescent deliveries was 37.22%. This finding is consistent with previous studies in Eastern Ethiopia 34.9% [6] and a study in the Dessie Amhara region of Ethiopia (32.5%) and is further supported by a study conducted in Turkey 28% [19]. However, the prevalence in this study is lower than the finding in southwest Uganda, 59.9% [20]. These contradictory results are due to small sample sizes, access to healthcare services, and socioeconomic conditions [18, 21].Factors such as poverty, low educational status, and inadequate family support are known to contribute to poor birth outcomes among adolescents [22].

On the other hand, among adolescent mothers, a significantly higher proportion of spontaneous vaginal deliveries was associated with an adverse birth outcome of 65%. This finding is in line with 61.8% of studies conducted in Jordan [23]. It has been suggested that adolescent pregnancy is associated with an increased risk of prolonged labor due to pelvic immaturity [24].

In this study, premature birth among newborns delivered by adolescent mothers was 8.83%. This study was in line with a study conducted in Sri Lanka at 7.6% [25] and in America at 8.3% [26]. Preterm birth is more common among younger mothers with short cervixes (less than 25 mm) and small uterine volumes [27]. In addition, the possible reason for this could also be that adolescents were exposed to high levels of psychological instability/stress due to cultural, social, and economic factors in their living environment [28].

In this study, adolescent mothers who had low haemoglobin levels (< 11 mg/dl) were 1.71 times more likely to have an adverse birth outcome than mothers whose hemoglobin levels were higher (> 11 mg/dl) (AOR = 1.71; 95% CI:1.04–2.81). This finding is consistent with similar studies reporting that low haemoglobin levels may increase adverse birth outcomes in adolescent mothers [29]. This may be due to the higher demands of adolescent mothers due to physical and physiological changes [30]. An important factor contributing to anaemia in adolescent mothers is dietary diversity [31].

According to this finding, low first-minute Apgar scores were 0.55 times more likely to be significantly associated with adverse neonatal outcomes in adolescent mothers than mothers whose mothers had an Apgar score greater than seven (AOR = 0.55; 95% CI: 0.45–0.66). This finding is in line with the study carried out in Slovakia [32] and Washington [33]. In addition, an Apgar score of less than 7 at the fifth minute was 1.11 times more likely Significantly associated with adverse neonatal outcomes in adolescent mothers than mothers who had an Apgar score of more than 7 (AOR = 1.11; 95% CI:1.00-1.22). The finding agrees with the study conducted in Washington [33], Ethiopia [6], Canada [34] and Cameroon [35]. Due to various sociodemographic, obstetric, and nutritional factors, low Apgar scores are more common in adolescent pregnancies [3].

Adolescent mothers who had eclampsia are 6.3 times more likely to have adverse birth outcomes than mothers without eclampsia (AOR = 6.3; 95% CI 1.74–22.75). This result is consistent with studies in Ethiopia [6], Sub-Saharan Africa [36], Cameroon [35] and Egypt [37]. This may be due to poor or lack of prenatal care, as poorer, less educated, and unemployed adolescent mothers had the major predisposition to eclampsia among adolescents in Ghana [38].

This study shows that adolescent mothers who underwent labor induction had a significantly higher (3 times) risk of experiencing an adverse neonatal outcome than those who did not undergo induction, comparable to studies in Taiwan [39].

According to this study, adolescent mothers with normal nutritional status had about 90% lower odds of experiencing adverse birth outcomes compared to those who had moderate acute malnutrition (AOR = 0.107, 95% CI 0.018–0.63), which is consistent with a study conducted in Butajira [40] and Ghana Ashanti [41]. This may be due to low socio-economic and educational status, leading to low health awareness and nutritional status, which may increase the risk of neonatal birth.

### Strengths and limitations of the study

The research is persuasive, among other factors, as it encompasses four major public hospitals in eastern Ethiopia. Nevertheless, the drawbacks stem from the exclusion of private maternity hospitals, the approach of retrospective data gathering, and potential bias arising from insufficient details on unfavourable birth outcomes.

## Conclusion

The overall result of the study shows that a high prevalence of newborns from adolescent mothers has an adverse birth outcome. Low birth weight, poor maternal nutrition, low haemoglobin level, and having eclampsia, induced during labor were factors significantly associated with adverse birth outcomes. Addressing factors like maternal nutrition, access to prenatal care, and proper delivery management could help reduce these adverse outcomes and improve overall maternal and neonatal health for adolescent mothers.

## Supplementary Information

Supplementary Material 1.

## Data Availability

No datasets were generated or analysed during the current study.

## Abbreviations

ANC: Antenatal care
AOR: Adjusted odds ratio
APGAR: Appearance, Pulse, Grimace, Activity, Respiration
APH: Antepartum haemorrhage
COR: Crude odds ratio
NICU: Neonatal Intensive Care Unit
